# Complete Genome Sequence of a Rat Hepatitis E Virus Strain Isolated in the United States

**DOI:** 10.1128/genomeA.01096-14

**Published:** 2014-11-06

**Authors:** Yannick Debing, Suzanne U. Emerson, Robert H. Purcell, Johan Neyts, Kai Dallmeier

**Affiliations:** a Rega Institute for Medical Research, Department of Microbiology and Immunology, KU Leuven, Leuven, Belgium; b Molecular Hepatitis and Hepatitis Viruses Sections, Laboratory of Infectious Diseases, National Institute of Allergy and Infectious Diseases, National Institutes of Health, Bethesda, Maryland, USA

## Abstract

Hepatitis E virus is a common cause of acute hepatitis in humans. Related viruses have been isolated from multiple animal species, including rats, but their impact on human health is unclear. We present the first full-length genome sequence of a rat hepatitis E virus strain isolated in the United States (LA-B350).

## GENOME ANNOUNCEMENT

Hepatitis E virus (HEV) is a positive sense, single-stranded RNA virus and is a major cause of acute hepatitis worldwide (1). Severe infections with high mortality may occur in pregnant women and chronic infections have been described in immunocompromised patients (2). HEV belongs to the *Hepeviridae* family that also comprises several related viruses isolated from different animals, including rats, ferrets, bats, rabbits, chickens, and teleost fish (3). The epidemiological aspects and public health impact of these animal viruses are unclear to date, as are those for rat HEV. At present, 10 full-length rat HEV genomes are available, including sequences isolated from Germany, Indonesia, and Vietnam (4). Rat HEV has also been detected in other regions, such as the United States, Denmark, and China (5–8), but no full-length sequences have been determined for these strains yet.

Here we present the first full-length rat HEV sequence isolated in the United States. The virus was originally isolated from rats trapped in urban Los Angeles, and then passaged twice in Sprague-Dawley rats and once in an athymic nude hooded rat (B350), as reported (5). Total RNA was extracted from 100 µL of a 10% liver homogenate from rat B350 with the Qiagen RNeasy kit (Hilden, Germany) and viral RNA was amplified using the One-Step reverse transcription (RT)-PCR kit (Qiagen) and KAPA Hi-Fi HotStart ReadyMix PCR kit (Kapa Biosystems, Wilmington, MA) subsequently. Primers were designed using relatively conserved regions in an alignment of known rat HEV sequences and strain-specific sequences as they became available. Fragments were gel purified, cloned into the pJet1.2 vector using the CloneJET PCR cloning kit (Thermo Fisher Scientific, Waltham, MA), and sequenced with the BigDye Terminator v. 3.1 cycle sequencing kit (Life Technologies, Carlsbad, CA). Sequences were assembled and manually edited to obtain a complete consensus sequence (9).

The genome of this particular rat HEV strain, designated LA-B350, is 6,942 nucleotides (nt) in length [excluding the poly(A)-tail] and encodes the expected open reading frame 1 (ORF1) (1,634 amino acids [aa]), ORF2 (644 aa), ORF3 (102 aa), and also the ORF4 (183 aa) that seems to be conserved in rat and ferret HEV (4). The highest nucleotide homology was found with the Indonesian ratESOLO-006SF strain (87.8%) and the German rat/Mu09/0434/DEU/2010 strain (87.6%), which have been provisionally classified into genetic group 1. This lineage also includes all other known German strains (4). Phylogenetic analyses of the full-length LA-B350 confirmed its classification into the first genetic group (designated G1). Sequence variation observed in the cloned partial cDNAs was on average 2 × 10^–3^, and may thus be slightly higher than expected for errors introduced by reverse transcription (range of 10^–4^ to 10^–5^) (10) and PCR (approximately 10^–4^) (http://www.qiagen.com/media/ebooks/Maximising_PCR_and_RT_PCR/index.htm), suggesting that LA-B350 may exist as a diverse viral quasispecies, as was described for human HEV as well (11).

In conclusion, the availability of the first U.S. rat HEV genome may help in further elucidating rat HEV epidemiology and its possible impact on human health.

### Nucleotide sequence accession number.

The complete genome of rat HEV strain LA-B350 can be accessed under the GenBank accession no. KM516906.
